# The Intra-S Checkpoint Responses to DNA Damage

**DOI:** 10.3390/genes8020074

**Published:** 2017-02-17

**Authors:** Divya Ramalingam Iyer, Nicholas Rhind

**Affiliations:** Department of Biochemistry & Molecular Pharmacology, University of Massachusetts Medical School, Worcester, MA 01605, USA; nick.rhind@umassmed.edu

**Keywords:** DNA damage, intra-S checkpoint, ATR, Chk1, fork stability, origin regulation

## Abstract

Faithful duplication of the genome is a challenge because DNA is susceptible to damage by a number of intrinsic and extrinsic genotoxins, such as free radicals and UV light. Cells activate the intra-S checkpoint in response to damage during S phase to protect genomic integrity and ensure replication fidelity. The checkpoint prevents genomic instability mainly by regulating origin firing, fork progression, and transcription of G1/S genes in response to DNA damage. Several studies hint that regulation of forks is perhaps the most critical function of the intra-S checkpoint. However, the exact role of the checkpoint at replication forks has remained elusive and controversial. Is the checkpoint required for fork stability, or fork restart, or to prevent fork reversal or fork collapse, or activate repair at replication forks? What are the factors that the checkpoint targets at stalled replication forks? In this review, we will discuss the various pathways activated by the intra-S checkpoint in response to damage to prevent genomic instability.

## 1. Introduction

Genetic material is constantly subject to insults by both intrinsic and extrinsic factors [1,2]. Genetic aberrations can also arise during replication of complex sequences that contain secondary structures or repeats [3,4,5]. DNA damage checkpoints safeguard the genome against these insults and ensure its faithful transmission across generations. Once activated, these checkpoints block cell cycle progression and ensure that the DNA is fully repaired before allowing progression to the next phase of the cell cycle [6]. However, even though the cell has checkpoints and repair pathways dedicated to DNA damage repair in G1, it is impossible to guarantee that cells will enter S phase with a perfect template, therefore the cell must be prepared to encounter damaged DNA during S phase [7,8,9]. In this review, we will discuss the various tactics employed by the intra-S checkpoint to minimize the casualties of S-phase DNA damage.

## 2. Source of Damage

### 2.1. Intrinsic Sources of Damage

DNA can be damaged by numerous intrinsic and extrinsic factors [1,2]. Intrinsic factors include reactive oxygen species (ROS) generated as a by-product of cellular metabolism, which can cause oxidative damage to DNA. Other toxic metabolites include reactive aldehydes generated via alcohol metabolism, which can crosslink DNA [10,11]. Apart from toxic by-products of metabolism, ribonucleotides can pose a threat, too [12]. Despite the specificity of DNA polymerases for deoxyribonucleotides over ribonucleotides, recent studies have shown that more than 10,000 ribonucleotides may be incorporated into the *Saccharomyces cerevisiae* genome during replication and can cause genomic stress if not actively removed [13,14]. In unperturbed cells, ribonucleotides are removed from the genome using a combination of ribonuclease H (RNaseH) activity and post-replication repair pathways. Replication stress can also be caused be intrinsically difficult to replicate sequences in the genome, such as G-quadruplexes and repeats, which can lead to replication fork slippage and chromosomal breaks [3,4,15]. Another natural impediment to the replication fork is the transcriptional machinery. Collision between the replication and the transcription machinery leads to fork stalling, R-loop formation, and topological stress, which may trigger DNA damage and recombination [16,17]. Cells have active mechanisms to constrain the deleterious effects of all these aberrations, so as to curtail their impact on the genome.

### 2.2. Extrinsic Sources of Damage

Extrinsic factors that damage DNA include ultra-violet light (UV) and ionizing radiation (IR), and chemicals such as methyl-methane sulfonate (MMS), mitomycin C, cisplatin, psoralen, camptothecin (CPT), and etoposide, to list a few of the well-known DNA damaging agents. These damaging agents cause different kinds of lesions, from simple alkylation of bases by MMS, to the more complex pyrimidine dimers by UV, topoisomerase-DNA covalent complexes by CPT, and inter-strand and intra-strand crosslinks by cisplatin and psoralen [18,19,20,21]. Cells have evolved various pathways to specifically detect and repair different kinds of lesions. The repair pathways include base excision repair (BER), which targets modified bases, nucleotide excision repair (NER) pathway, which targets more complex modifications such as pyrimidine dimers. Inter-strand crosslinks are repaired using inter-strand crosslink repair pathway, which involves a combination of repair pathways consisting of NER, homologous recombination (HR), TLS (translesion synthesis), and Fanconi anemia (FA) repair pathways. Finally, double strand breaks (DSB) are repaired by non-homologous end-joining (NHEJ) and HR pathways [1,8,18,21,22,23,24,25,26].

## 3. The Intra-S Checkpoint

Despite having specific repair pathways dedicated to each kind of DNA lesion, the cell relies on a single checkpoint to mediate the DNA damage response during S phase. The cell has two main checkpoint kinases, Ataxia Telangiectasia Mutated (ATM) and ATM and Rad3-related (ATR), both of which are critical for maintaining genomic integrity. Of the two, ATR is the more crucial mediator of intra-S checkpoint responses since it is activated in response to diverse lesions. ATM (Tel1 in budding and fission yeast) mainly responds to double strand breaks, while ATR (Mec1 in budding yeast, Rad3 in fission yeast) is activated in response to a variety of genotoxins such as UV, MMS, hydroxyurea (HU), aphidicolin, and psoralen. ATR also functions in every unperturbed S phase, where it regulates origin firing [27,28,29,30,31,32]. Since several different pathways activate ATR in response to diverse lesions, it has been suggested that the checkpoint is activated by a common repair intermediate [33,34,35,36,37,38,39].

## 4. Detection of Lesion During S Phase

The first step key to all repair pathways is the detection of the lesion itself. Detection of a lesion can be a challenge in the vast pool of undamaged template [9,23]. Furthermore, individual damaged bases must be detected in the context of DNA complexed with protein and condensed into chromatin [40]. Depending on the severity of lesions, certain aberrations may be detected only during the act of replication itself. The replication fork is a sensitive detector of lesions, since it has to interact with every base in the genome during replication. Several studies have shown that lesions caused by UV and MMS activate the checkpoint only during S phase [41,42,43,44,45,46]. Studies in *S. cerevisiae* have shown that, if replication initiation is blocked using conditional alleles of initiation factors such as Cdc6, or Cdc45, or Cdc7, then cells undergo nuclear division without replicating DNA or activating the checkpoint even when treated with 0.033% MMS, demonstrating that this level of damage is not recognized outside of S phase [43]. However, during S phase, as little as 0.005% MMS is sufficient to activate the checkpoint, suggesting that the replication fork is a highly sensitive and efficient activator of the checkpoint [43]. Similarly, in *Xenopus* extracts, prevention of replication by addition of geminin blocks checkpoint activation in response to UV and MMS induced lesions [44,45]. In human cells too, ATR activation in response to UV requires replication [47].

UV- and MMS-induced lesions at high concentrations can activate the DNA damage checkpoint outside S phase. Such activation relies on repair pathways such as BER in the case of MMS-induced lesions and NER in the case of UV-induced lesions to generate intermediate structures capable of activating the checkpoint [48,49,50,51,52,53]. Thus, the checkpoint can be activated by stalled replication forks as well as intermediate structures generated by repair pathways in response to diverse lesions caused by different agents such as UV, MMS, and aphidicolin [34].

## 5. Intra S-Checkpoint Activation

### 5.1. The Structure Necessary for Checkpoint Activation

The fact that ATR is activated in response to different kinds of genotoxins suggests that the activation might occur not through recognition of damage itself but a common intermediate generated in response to any lesion that perturbs replication. Several studies indicate that the common intermediate necessary for checkpoint activation is replication protein A (RPA)–single-stranded DNA (ssDNA) complex [35,36,54,55,56,57,58]. Replicative polymerases tend to stall in response to lesions while the helicase continues to unwind the DNA ahead of the lesion. Such uncoupling of the helicase and the polymerase leads to generation of ssDNA, which gets coated with the ssDNA binding protein RPA [35,36,57,58]. This common intermediate comprised of stalled replicative polymerase allows for a simple mode of checkpoint activation by diverse lesions [35,36]. In the cases of double-strand breaks and inter-strand crosslinks—which do not directly produce ssDNA—lesion processing creates ssDNA, as described below.

### 5.2. The Factors Necessary for Checkpoint Activation

Several studies have shown that RPA coated ssDNA is essential for activation of the S-phase checkpoint kinase, ATR [54,57,58,59,60]. ATR is a highly-conserved checkpoint kinase, which responds to various kinds of lesions that block DNA replication [61,62]. RPA bound ssDNA interacts with ATR-interacting protein (ATRIP) (Ddc2 in budding yeast, Rad26 in fission yeast), which binds ATR, leading to its recruitment to sites of DNA damage (Table 1) [57,60,63,64].

**Figure 1:**
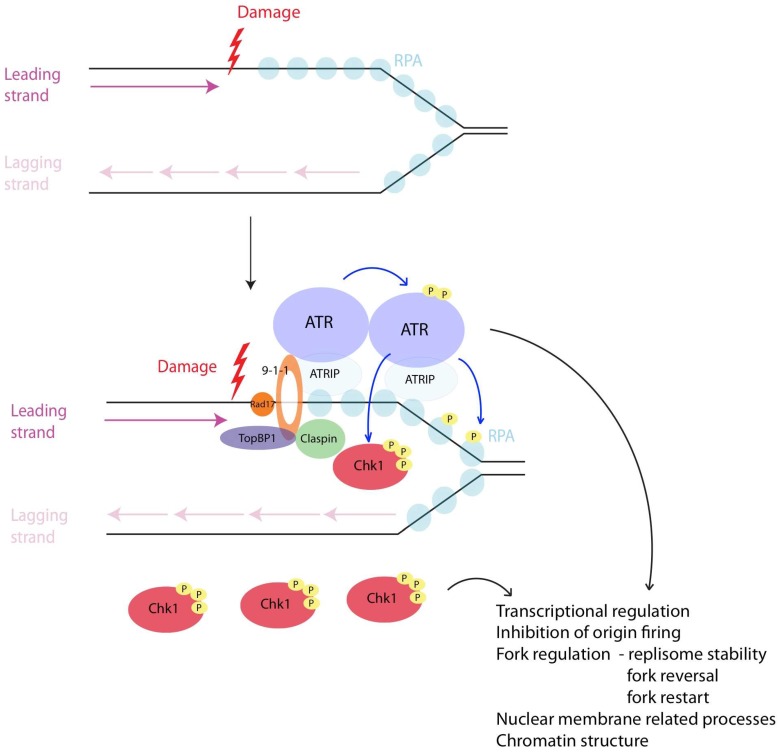
Intra-S checkpoint activation. Fig1 depicts how a stalled fork generates RPA-ssDNA which subsequently recruits ATR-ATRIP, Rad17/9-1-1, TopBP1 leading to ATR activation. Rad17/9-1-1 complex further recruits adaptors like Claspin which leads to transduction of the signal to the effector kinase Chk1. Chk1 and ATR phosphorylate a wide range of substrates affecting several aspects of cellular physiology in response to damage such as transcription, replication kinetics, modulation of nuclear membrane processes and alteration of chromatin structure.

The stalled fork junction composed of ssDNA-RPA complex and dsDNA further recruits Rad17-RFC complex, which loads a trimeric ring-shaped complex Rad9-Rad1-Hus1 (9-1-1) at sites of damage, although it is unclear if Rad17-RFC recognizes the 3'ds/ssDNA junction, perhaps after polymerase release or a 5'ds/ssDNA junction, which would be created by repriming ahead of a stalled polymerase on either the leading or lagging strand (Figure 1) [65,66].

9-1-1 complex in turn recruits DNA topoisomerase II binding protein 1 (TopBP1) (Dpb11 in budding yeast, Cut5 in fission yeast), which further stimulates ATR activity [66,67,68,69,70,71,72,73,74,75]. Rad17-RCF and 9-1-1, together with regulators Claspin (Mrc1 in budding and fission yeast) and Tim/Tipin (Tof1/Csm3 in budding yeast, Swi1/Swi3 in fission yeast), are essential for activation of checkpoint kinase 1 (Chk1), which is the main target of ATR and the effector kinase in the checkpoint pathway in metazoa (Figure 1) [76,77,78,79,80,81,82,83,84,85].

### 5.3. Downstream Effectors of Checkpoint Activation

ATR and ATM activate two effector kinases, Chk1 and Chk2, in response to damage to relay the checkpoint signal across the cell. While ATR and ATM mainly target substrates at the chromatin close to the site of lesion, the effector kinases freely diffuse and transduce the signal to distant substrates [86,87,88,89,90,91,92]. In mammals, Chk1 and Chk2 play overlapping roles. Although Chk1 is primarily activated by ATR in response to various kinds of lesions and Chk2 by ATM in response to DSBs, there is substantial cross-talk between the two pathways making it difficult to unambiguously assign Chk1 and Chk2 to a single checkpoint pathway [7,80,93,94,95,96,97,98,99]. The roles played by Chk1 and Chk2 also vary substantially across species [100]. In budding and fission yeasts, Rad53 and Cds1 are homologs of mammalian Chk2, respectively. However, they are functionally equivalent to mammalian Chk1. In budding yeast Rad53 is required for the intra-S checkpoint as well as G2/M checkpoint responses, while Cds1 in fission yeast acts only during S phase [61,101,102,103,104].

Inter-strand crosslinks also activate ATR, even though they do not generate RPA-ssDNA in the canonical way by uncoupling helicase and the polymerase. To activate ATR, inter-strand crosslinks rely on the FA pathway. Processing of the inter-strand crosslink by the FA pathway leads to generation of ssDNA-RPA, which in turn activates ATR. Inhibition of FA pathway or depletion of FANCD2 greatly diminishes Chk1 activation in response to inter-strand crosslinks [105,106].

## 6. Strength of Checkpoint Activation

Replication initiation involves melting of DNA, which produces RPA-coated ssDNA, the structure necessary for checkpoint activation. Therefore, one complication of checkpoint activation via RPA-ssDNA complex is that it is a common intermediate generated even during an unperturbed S phase. Several studies indicate that the checkpoint functions in every S phase even in the absence of damage. The importance of this function is suggested by the fact that inhibition of Chk1 during unperturbed S phase leads to unrestrained origin firing, which is detrimental to genomic stability [27,28,29,30,31,32]. The effect of the checkpoint during unperturbed replication can also be seen in *Xenopus* extracts, where the rate of replication decreases with increasing concentration of template in a checkpoint-dependent manner [27,107]. Therefore, it appears that the ssDNA-RPA structures of many unperturbed replication forks are capable of collectively activating the checkpoint, even in the absence of damage.

Even though the checkpoint is activated in every S phase, there is a quantitative difference between level of activation during an unperturbed S phase and level required to be induced by DNA damage to activate a full-strength checkpoint response. The level of Chk1 activation is tightly correlated with the amount of ssDNA generated. In the presence of fork stalling lesions the helicase becomes uncoupled from the polymerase leading to generation of longer stretches of ssDNA than present in an unperturbed fork [58]. The excess ssDNA-RPA complex formed in response to DNA damage leads to robust activation of the checkpoint. Titration experiments with plasmids of varying sizes in *Xenopus* extracts show that the amount of ssDNA generated determines the strength of Chk1 activation [58]. Along similar lines, the number of active forks determine the activation of Rad53 in response to DNA damage in *S. cerevisiae* [108].

Although double strand breaks primarily activate ATM, resection of their ends leads to ssDNA generation leading to subsequent activation of ATR [95,96,109,110,111,112]. The strength of checkpoint activation and subsequent cell cycle delay in response to DSB is regulated by both the number of DSBs generated and the amount of ssDNA generated at each DSB [112,113,114]. Thus, the checkpoint activation can be quantitatively modulated by the amount of ssDNA generated in response to different kinds of lesions.

## 7. Downstream Targets

Unlike ATR, which mainly phosphorylates substrates on chromatin, the S-phase effector kinases transduce the signal to many targets across the cell [86,87,88,89,90,91,92]. Activation of Chk1 in metazoans and Rad53 and Cds1 in yeast in response to replication stress leads to regulation of replication kinetics via inhibition of origin firing and regulation of replication forks, and to transcriptional reprogramming.

## 8. Transcriptional Regulation by the Checkpoint

### 8.1. G1/S Regulation

In both mammals and yeast, the S-phase checkpoint maintains transcription of G1/S genes, which are normally turned off as the cells progress through S phase. In mammals, Chk1 regulates the E2F family of transcription factors, whose targets are involved in DNA metabolic processes and DNA repair. Repression of E2F targets during a replication stress response generates further DNA damage signals and hampers cell survival, demonstrating the importance for checkpoint-dependent maintenance of their expression during replication stress [115]. Along similar lines, expression of G1/S genes are maintained in response to replication stress in *S. cerevisiae* and *S. pombe*. Mlu1-box binding factor (MBF) induces the expression of G1/S transition genes, which are inactivated by the Nrm1 transcriptional repressor as cells progress through S phase. Both Rad53 in *S. cerevisiae* and Cds1 in *S. pombe* phosphorylate and inactivate Nrm1 to maintain expression of G1/S genes in response to replication stress [116,117,118,119].

In *S. cerevisiae*, the importance of transcriptional responses activated by the checkpoint is not clear. Several independent studies have shown that Rad53 maintains S-phase transcription of several hundreds to thousands of genes in response to damage. However, since these genes constitute the entire G1/S regulon, most of the upregulated transcripts do not encode for DNA repair proteins or proteins whose deletion induces sensitivity in response to DNA damage [120,121,122,123]. Furthermore, Tercero et al., have shown that new protein synthesis is not necessary to resume fork synthesis or maintain cell viability when released from a hydroxyurea (HU) arrest [43]. However, it is unclear whether new protein synthesis is dispensable when forks actively encounter fork-stalling lesions as in the case of MMS treatment during S phase. In *S. pombe*, the maintenance of specific G1/S transcripts has been shown to contribute to resistance to replication stress [117,124,125].

### 8.2. RNR Regulation

In addition to maintenance of S-phase transcription in response to damage, the checkpoint also regulates RNR (ribonucleotide reductase) activity, which is required for deoxynucleotide triphosphate (dNTP) synthesis [126,127,128,129,130,131,132,133,134,135,136,137,138,139,140,141,142,143]. In budding yeast, activated Rad53 induces RNR expression by phosphorylating the Dun1 kinase, which in turn phosphorylates and inactivates Rfx1 (aka Crt1). Rfx1 transcriptionally represses RNR genes by recruiting Tup1-Ssn6, thus its inactivation strongly induces RNR expression [126]. In a similar manner, fission yeast and mammalian cells also upregulate transcription of RNR genes in a checkpoint dependent manner [130,131].

Apart from transcriptional regulation, RNR activity is also modulated through regulation of its localization as well as by small protein inhibitors. In budding yeast, Dun1 phosphorylates Dif1, a protein that sequesters Rnr2-Rnr4 subunits in the nucleus and thus prevents the subunits from forming an active complex together with the Rnr1 subunit in the cytoplasm. Phosphorylation of Dif1 triggers its degradation leading to release of Rnr2-Rnr4 to the cytoplasm [135,136]. Dun1 also phosphorylates Sml1, an inhibitor of Rnr1 and targets it for degradation [137]. In fission yeast, the checkpoint targets Spd1 for degradation, which affects both localization and activity of RNR subunits [139,140,141,142,143]. Furthermore, a related protein Spd2 may also affect RNR regulation in fission yeast [144]. A recent study in mammalian cells has identified Inositol 1,4,5-triphosphate (IP_3_) receptor binding protein released with IP_3_ (IRBIT) as an inhibitor of RNR activity, however its regulation by the checkpoint is yet to be determined [145]. Thus, using multiple mechanisms dNTP production is increased in response to damage, which greatly improves cell viability [128,139,146]. Of the three model organisms, budding yeast shows the most dramatic increase in dNTP levels in response to damage, which perhaps explains why *S. cerevisiae* is resistant to much higher concentrations of HU than other organisms [138,139,146].

## 9. Regulation of Replication Kinetics by the Checkpoint

Slowing of replication in response to DNA damage has been documented for more than half a century [147,148,149,150]. The initial hints of checkpoint regulation of replication slowing came from Ataxia Telangiectasia (AT) patients, characterized by hypersensitivity to IR. Cells from AT patients fail to slow replication in response to IR, a characteristic termed ‘radio-resistant DNA synthesis’ [151,152,153,154]. AT patients suffer from severe developmental defects and are highly predisposed to developing cancer [155,156]. The symptoms of AT patients highlight the importance of checkpoint regulated slowing of replication in response to damage. Later studies in *S. cerevisiae* and *S. pombe* showed that slowing of S phase is an evolutionarily conserved mechanism in response to DNA damage [102,103,157]. This bulk slowing of replication is achieved through a combination of inhibition of origin firing and regulation of fork progression.

## 10. Inhibition of Origin Firing

Replication of the genome occurs in a temporally ordered manner with different parts of the genome replicating at specific times in S phase [158]. In the presence of damage, the early origins fire regardless of the presence of lesions, since the forks established by early origins are the ones which sense the lesions and activate the checkpoint. Once the checkpoint is activated, it suppresses firing of late origins [159,160,161,162,163,164,165,166,167,168]. In *S. cerevisiae*, Rad53 phosphorylates the origin activation factors Sld3 and Dbf4 in response to replication stress to prevent subsequent origin firing [169,170]. Sld3 is a replication-fork assembly factor required during early steps of replication initiation; Dbf4 is the regulatory subunit of Dbf4-dependent kinase (DDK), which is required for origin firing and fork progression [171,172,173]. In mammals, Chk1 targets multiple substrates to block origin firing. In response to IR, Chk1 phosphorylates Cdc25A, targeting it for ubiquitin-mediated degradation. Cdc25A is a phosphatase necessary for Cdk2-CyclinE activity, which is required for binding of Cdc45 to the pre-replicative complex (pre-RC) and initiating replication [30,163]. Chk1 also phosphorylates Treslin, the metazoan homolog of Sld3, to prevent loading of Cdc45 onto chromatin [174]. Further studies in *Xenopus* and mammalian cells suggest that Chk1 also targets DDK in response to UVC and etoposide treatments [59,175,176]. Inhibition of origin firing prevents new forks from encountering damage and stalling. Although reduction in origin firing leads to slowing of replication, which is critical, it does not significantly contribute to maintenance of cell viability, at least not in *S. cerevisiae* [43].

### 10.1. Activation of Dormant Origins

Although checkpoint activation inhibits origin firing globally, several reports suggest that it might allow dormant origins to fire locally in response to replication stress [177,178]. Cells license origins during G1 phase of the cell cycle and activate them throughout S phase [179,180,181,182]. In an unperturbed S phase, a cell fires only about 10% of its licensed origins [178,183,184]. Most of the remaining origins are licensed but not fired and hence referred to as dormant origins. During unperturbed replication, dormant origins are passively replicated. However, in the event of replication stress, forks from early origins stall and the dormant origins remain un-replicated. Under such conditions, the dormant origins fire and help complete replication in the vicinity of stalled forks and thereby mitigate the consequences of fork stalling. Reduction of dormant origin firing via depletion of mini-chromosome maintenance (MCM) complex makes the cell hypersensitive to replication perturbation, highlighting the importance of dormant origins [183,185,186,187]. At this point, it is unclear how the checkpoint could suppress origin firing globally but permit activation of dormant origins in response to replication stress. A possible explanation is that the checkpoint reduces origin firing globally, but that even so dormant origin firing increases due to the reduction in passive replication [177,178].

## 11. Fork Regulation

### 11.1. Importance of Fork Regulation

Several studies suggest that the regulation of replication forks in response to replication stress is the crucial function of the intra-S checkpoint. The first hint of the importance of fork regulation came from the discovery of a separation of function mutant in budding yeast called *mec1-100* [188]. *mec1-100* cells cannot suppress origin firing in response to stress, but are not hypersensitive to MMS, unlike *mec1Δ* cells [43,188]. Presumably fork regulation is intact in *mec1-100*, hinting that fork regulation is more critical for cell viability in response to MMS than origin firing inhibition. Consistent with this conclusion, Tercero et al. have shown that forks progress slowly but stably in *mec1-100* to complete replication in response to MMS [43]. In contrast, in *mec1Δ* and *rad53Δ* cells treated with HU or MMS, forks collapse irreversibly leading to large stretches of un-replicated DNA [42,189]. Experiments in which Rad53 expression is suppressed during HU treatment but induced after release from HU arrest show that the checkpoint is necessary at the time of fork stalling to maintain the replication fork in a restart competent manner. Expression of Rad53 after release from HU arrest is not sufficient to maintain viability [43]. Along similar lines in mammals, *ATR-/-* and *CHK1-/-* are embryonic lethal in mice and inactivation of ATR during replication stress greatly hampers fork progression and cell viability [190,191,192]. Collectively, these studies suggest that the checkpoint is essential for preventing fork collapse in response to replication stress. The mechanism by which the checkpoint accomplishes fork stabilization and maintains cell viability is not understood.

### 11.2. Regulation of Number of Forks

In response to replication stress, suppression of late firing origins limits the generation of an excess number of stalled forks. Unrestrained firing of origins in the presence of replication stress might overwhelm the capacity of the checkpoint to attenuate the consequences of stalled forks. Supporting this idea, Toledo et al. observed that in the absence of ATR activity, excess firing of origins in response to HU depletes the nuclear pool of RPA leading to DSB generation [193]. Therefore, the critical role of the checkpoint may not be to regulate the fork per se but to curtail origin firing in response to replication stress so as to avoid generation of an excess number of stalled forks. However, it is yet to be determined whether replication forks from ATR inhibited cells supplemented with excess RPA are capable of stably progressing and completing replication when released from HU arrest. Furthermore, HU treatment in the absence of a checkpoint leads to excessive unwinding and generation of longer stretches of ssDNA as compared to cells in which the checkpoint activity is intact [194]. Therefore, RPA may have a more critical role under excessive unwinding, as seen in checkpoint mutants, than in wild-type cells.

### 11.3. Maintenance of Replisome Stability

**Figure 2:**
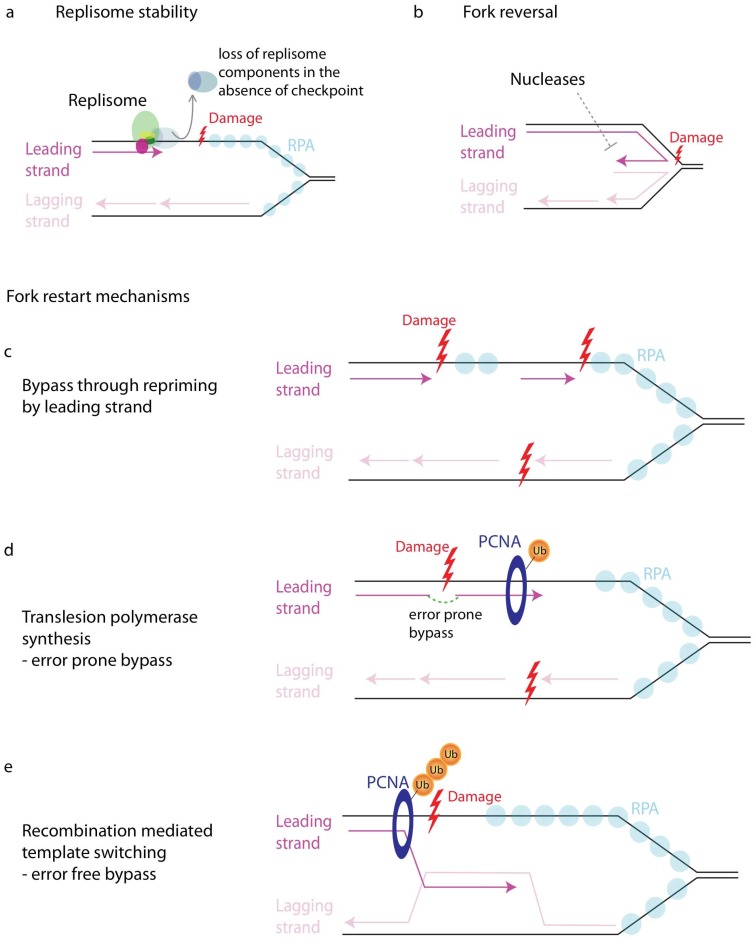
Regulation of forks in response to damage. (**a**) Replisome stability pertains to stable association of replisome components; (**b**) Fork reversal in response to damage, wherein the leading strand anneals with the lagging strand to form a four-way structure. Fork reversal is opposed by nucleases such as Exo1, Dna2; (**c**) Downstream repriming. Leading strand can bypass damage by repriming downstream of the stalled fork; (**d**) Translesion polymerase based synthesis. A stalled fork can bypass damage by recruiting translesion ploymerase in an error prone manner. Recruitment of translesion polymerase requires mono-ubiquitination of PCNA; (**e**) Template switching. A stalled fork can bypass damage by using the lagging strand as a template instead of the damaged parental strand. Template switching requires poly-ubiquitination of PCNA.

The most controversial role of the checkpoint at stalled forks is the maintenance of replisome stability [195,196]. Replisome stability refers to the physical association of the replisome factors with the stalled replication fork (Figure 2a).

Several chromatin immuno-precipitation (ChIP) studies done in budding yeast have suggested that, in response to HU, polymerases and helicases tend to dissociate from the stalled fork in the absence of an active checkpoint [197,198,199,200,201]. Similarly, studies in *Xenopus* and mammalian cells have shown that several components of the replisome are lost from forks stalled in response to prolonged treatment with aphidicolin in the absence of ATR [202,203,204]. However, contrary to these studies, De Piccoli et al. have shown—using genome-wide ChIP-seq—that the replisome components remain stably associated with forks stalled in HU even in the absence of Rad53 or Mec1 in budding yeast [205]. Perhaps the discrepancy between these reports can be explained by the differences between their ChIP assays. The former focused on early origins with ChIP PCR probes designed at close proximity to the early origins as opposed to genome-wide ChIP-seq by the latter, which gives a more comprehensive picture. The latter work shows that, in the absence of checkpoint, forks from early origins continue to replicate longer and hence stall replisome components further downstream than they would in wild-type cells [205]. Thus, by ChIP-PCR with probes designed at close proximity to the early origins, the replisome components appear to be intact in wild-type and depleted in the checkpoint mutant [205]. However, at this point, it remains a matter of debate whether the checkpoint affects the physical association of the replisome components or only regulates their functionality in response to replication stress [196].

Most studies trying to understand the role of checkpoint in maintaining replisome stability have focused on forks stalled for a prolonged duration (20 to 60 min) in response to HU arrest. Stalling forks in the order of tens of minutes in response to HU might be biologically very different than fork pausing briefly in response to MMS-induced lesions. It is not clear whether stability of the replisome components is affected if the fork stalls are short-lived as compared to that in a HU arrest. Therefore, the mechanism by which Rad53 allows the forks to progress slowly but stably and complete replication of the whole genome in response to MMS remains unclear.

### 11.4. Fork Reversal

Regardless of whether the checkpoint affects replisome stability or not, it prevents accumulation of pathological structures at stalled replication forks. *rad53* mutants accumulate structures similar to those obtained by destabilizing replisome components as monitored by 2D gels [189]. Similarly, electron microscopy (EM) studies have shown that HU treatment of *rad53Δ* cells leads to excessive unwinding and generation of longer stretches of ssDNA as compared to wild-type cells [194]. Furthermore, *rad53Δ* cells accumulate reversed forks wherein the leading strand is unwound and anneals with the lagging strand to form a four-way structure (Figure 2b) [194,199]. Whether reversed forks are a pathological structure or productive repair intermediates is uncertain. In yeast, fork reversal is mainly observed in the absence of checkpoint and therefore appears to be pathological. However, in metazoans, fork reversal appears to be a part of DNA damage tolerance mechanism [206]. Chaudhuri et al. have shown that in mammalian cells, *Xenopus* extracts, and yeast cells, low doses of CPT treatment lead to fork reversal. In mammals, reversal of forks is mediated by poly (ADP-ribose) polymerase 1 (PARP1) [207]. Depletion of PARP1 prevents fork reversal and leads to double strand break formation [207]. Furthermore, Rad51 dependent fork reversal is seen in human cells in response to a variety of genotoxins [208]. Thus, in mammals, fork reversal appears to play a protective role. However, in the absence of checkpoint, nucleases such as Mus81 and Slx4 can improperly process reversed forks leading to genomic instability [190,209,210]. Thus, fork reversal itself may not be pathological, but its regulation by the checkpoint may prevent deleterious outcomes. In vitro biochemical studies have identified several helicases and translocases such as Rad54, WRN, BLM, HLTF, FANCM, FBH1, SMARCAL1, and ZRANB3 capable of regressing forks [211,212,213,214,215,216,217,218,219,220,221,222,223]. However, of all these factors, only Rad51 and FBH1 have been shown to be required for fork regression in vivo [208,221]. Furthermore, how helicases and translocases may be regulated by the checkpoint at stalled forks is not known.

### 11.5. Regulation of Nucleases

There is mounting evidence that the checkpoint plays a role in protecting forks from aberrant activity of nucleases. Support for this idea comes from Segurado and Diffley, 2008 work, which shows that deletion of *EXO1* rescues *rad53Δ* sensitivity to several genotoxins like UV, MMS, and IR all except HU [224]. Phospho-proteomic screens have also identified Exo1 as a target of Rad53 and this phosphorylation has been shown to negatively regulate Exo1’s activity [88,225]. Furthermore, EM studies in budding yeast have shown that Exo1 creates ssDNA intermediates of reversed forks and drives fork collapse in the absence of Rad53 [199]. However, deletion of *EXO1* alone is not sufficient for fork stabilization. Forks are unable to restart when released from HU arrest even in a *rad53Δexo1Δ* background similar to *rad53Δ* [224]. Thus, Rad53 has Exo1-independent functions at maintaining fork integrity.

In fission yeast, Cds1 phosphorylates and activates Dna2, a helicase/nuclease, which prevents accumulation of reversed forks [226]. In human cells, DNA2 is involved in the processing and restart of reversed forks [227,228]. Thus, the checkpoint modulates fork reversal by activating or inhibiting nucleases.

### 11.6. Restart of Stalled Forks

The ultimate question of how the checkpoint restores progression of stalled forks beyond the lesion is just being uncovered. As mentioned above, stalled forks can undergo fork reversal even in the presence of checkpoint. In human cells, reversed forks are restarted in a RECQ1 and DNA2 dependent manner [227,229]. Mus81-Eme1, a structure specific endonuclease, is normally active only during mitosis due to the requirement of phosphorylation by CDK1 and Polo-like kinase 1 (Plk1) for activation [230,231,232]. However, several recent studies suggest that Mus81 could also play a role in fork restart mechanisms during S phase by creating double strand breaks and promoting recombination [233,234,235,236,237,238,239,240,241]. In human cells, fork cleavage and restart of stalled forks in S phase is governed by Mus81-Eme2, while the G2/M functions of Mus81 are guided by Mus81-Eme1 complex [234,236,237]. SMARCAL1 may also be an important candidate, as it possesses both fork reversal as well as fork restoration activities, and is regulated by ATR [190,212,213]. However, its exact function at stalled forks in vivo is yet to be determined.

In the case of stalled forks that have not reversed, restart or restoration of fork progression occurs mainly in three ways: by repriming (Figure 2c), by translesion-polymerase-based synthesis (TLS) (Figure 2d), or by template switching (Figure 2e) [242,243,244,245,246,247,248]. Lesions on the lagging strand can be easily bypassed due to the discontinuous nature of lagging-strand synthesis. However, lesions on the leading strand must be actively bypassed using various mechanisms in order to continue DNA synthesis. The first evidence that lesion bypass via repriming downstream could be employed in the case of leading strand comes from studies done in bacteria. Bacterial replisomes are capable of repriming and re-initiating replication in response to UV-induced lesions (Figure 2c) [249,250]. Recent discovery of similar activity by PrimPol in human cells shows that repriming downstream may be an evolutionarily conserved approach. PrimPol, which has primase as well as translesion polymerase activity, allows repriming of stalled forks in response to UV as well as dNTP depletion [251,252,253]. Furthermore, EM studies suggest that repriming activities on leading strand in response to UV occurs in budding yeast, too [254], although it must be via a distinct mechanism, because PrimPol is not conserved in yeast [255].

The TLS and template switching mechanisms of fork restart are regulated by ubiquitination of the proliferating cell nuclear antigen (PCNA) [256,257,258]. ssDNA generated in response to replication stress recruits Rad18, which, along with Rad6, monoubiquitinates PCNA at K164 [256,259]. Monubiquitination of PCNA allows recruitment of translesion polymerases, which have low fidelity, allowing the fork to replicate across damaged bases (Figure 2d) [260,261,262]. Although translesion polymerases permit replication across damaged template, the bypass occurs in an error prone manner. PCNA can also be polyubiquitinated at K164 by Rad5 along with Mms-Ubc13 [256,263,264]. Polyubiquitination of PCNA promotes template switching (Figure 2e) [265,266,267,268]. Template switching involves usage of the undamaged sister chromatid for bypass of lesions and usually occurs in an error free manner. Inhibition of polyubiquitination increases TLS-based mutations suggesting competition between TLS and template switching pathways [268]. SUMOylation at K164 of PCNA also affects template switching [269,270,271]. The exact role of polyubiquitination of PCNA and how it leads to recruitment of the recombination factors necessary for template switching are not known [242,245,258,272]. Regulation and crosstalk across various modifications on PCNA and the role of checkpoint in mediating lesion bypass are also poorly understood. Furthermore, PCNA functions as a trimmer at the replication fork. Therefore, at a single stalled fork, individual copies of PCNA may harbor different modifications and the trimmer collectively may regulate the mechanism of lesion bypass [242,245,247,272].

## 12. Conclusions

The intra-S checkpoint plays a crucial role in maintaining genomic stability in response to various kinds of DNA damage. The checkpoint maintains genomic stability primarily by regulating origin firing, fork progression, and G1/S transcription in response to DNA damage. Of the three, regulation of forks is perhaps the most critical function of the checkpoint but its mechanisms remain largely unclear and controversial. Important insight into the role of fork regulation comes from EM studies, which have helped us uncover the structural alterations observed at stalled forks, and from in vitro biochemical studies with fork components and artificial templates, which have allowed us to decipher their catalytic functions. However, how the fate of a stalled fork is determined by the interplay of various factors in vivo is unclear. Recent advances using new techniques such as iPOND (isolation of proteins on nascent DNA) have led to identification of new players at stalled forks [273,274,275,276]. Although the list of proteins associated with the stalled fork grows, their regulation by the checkpoint is yet to be elucidated. In the future, direct observation of the resolution of stalled forks, as well as the ability to monitor single molecules of protein in action at the fork, will be critical to furthering our understanding of checkpoint mediated stable progression of replication forks through damaged templates.

## Figures and Tables

**Table 1:** List of key proteins involved in intra-S checkpoint activation conserved across species.

Title 1	*S. cerevisiae*	*S. pombe*	Mammals
Checkpoint kinase	Mec1 Ddc2 Rad24	Rad3 Rad26 Rad17	ATR ATRIP Rad17
Sensors	Ddc1 Mec3 Rad17 Dpb11	Rad9 Hus1 Rad1 Cut5	Rad9 Hus1 Rad1 TopBP1
Adaptors	Mrc1 Tof1 Csm3	Mrc1 Swi1 Swi3	Claspin Tim Tipin
Effector kinase	Rad53	Cds1	Chk1
